# Corrosion Assessment of a Weathering Steel Bridge Structure after 30 Years of Service

**DOI:** 10.3390/ma14143788

**Published:** 2021-07-06

**Authors:** Agnieszka Królikowska, Leszek Komorowski, Izabela Kunce, Damian Wojda, Katarzyna Zacharuk, Urszula Paszek, Tomasz Wierzbicki, Katarzyna Bilewska

**Affiliations:** 1 Road and Bridge Research Institute, 03302 Warsaw, Poland; lkomorowski@ibdim.edu.pl (L.K.); ikunce@ibdim.edu.pl (I.K.); dwojda@ibdim.edu.pl (D.W.); kzacharuk@ibdim.edu.pl (K.Z.); upaszek@ibdim.edu.pl (U.P.); 2 Engineering Department, Helena Chodkowska University of Technology and Economics, 03302 Warsaw, Poland; rektorat@uth.edu.pl; 3 Łukasiewicz Research Network—Institute of Non-Ferrous Metals, 44101 Gliwice, Poland; katarzyna.bilewska@imn.gliwice.pl

**Keywords:** weathering steel, protective ability index, bridge inspection

## Abstract

The first steel with improved resistance towards atmospheric corrosion, the so-called weathering steel, was patented in the USA in 1933 and was initially used for coal railway cars, and after that, in building and bridge engineering. Weathering steels show higher corrosion resistance than carbon steels in many types of atmosphere due to their ability to form a compact, stable, adherent and protective patina during the time of exposure. Morphological evaluation of the appearance of the corrosion product layer, together with phase analysis of its components, can enable determination of the type of patina and the degree of protection of the steel. To support the visual assessment of a patina, a check based on the qualitative and quantitative phase analysis of its components may be carried out, and the PAI (Protective Ability Index) can be calculated. The estimation of the corrosion processes on original Polish-made weathering steel (12HNNbA) was carried out on a 30-year-old bridge in Poland. There are some structural problems within the deck derived not only from corrosion but also steel cracking, both inside and outside the boxes, at different heights. Fourteen representative samples of patina were analysed and their phase structures were determined by the X-ray powder diffraction method. The PAIs were determined and analysed.

## 1. Introduction

Weathering steels contain up to 2 wt.% of alloying elements such as Cr, Mn, Si, Ni, Cu, Al and Nb. The corrosion product layer with protective properties, the so-called “patina”, is typically produced during 3 to 7 years of exposure under suitable environmental conditions, including persistent wetting and drying atmospheric cycles. The rust layer is composed of different iron oxy-hydroxides that act as a barrier and provide the protective ability. Although the corrosion rate of weathering steel is less than 0.01 mm/year, engineering structures made from that type of structural material must be inspected periodically. However, visual assessment may be unreliable, as it is the result of individual judgement and is based on the experience of the inspector. To support the visual assessment, qualitative and quantitative analysis of the components of the patina may be carried out, and the PAI may be estimated.

The examined bridge is the biggest piece of engineering structure made of unpainted weathering steel in Poland. The “Defenders of Modlin 1939 Bridge” crosses the Vistula River near Zakroczym, 30 km north of Warsaw. The bridge was conceived as two independent structures, one for each direction of the S7 express route. The static system of the bridge is a continuous six-span beam, with a range of 75.0 m for the edge spans and 95.0 m for the intermediate spans. The total length of the bridge structure is 531.4 m. The 14.1 m wide structure has two traffic lanes, 3.5 m wide each. The span structure of the bridge consists of a steel box girder with a bridge deck in the form of an orthotropic plate with U-shaped closed ribs. The top plate of the orthotropic plate is 14 mm thick at the spans and 18 mm at the support sections. In the web and bottom flange, 14 mm thick plates are used as the basic plates; 18 and 22 mm thick plates are used near the intermediate supports. The spacing between the transverse frames formed by the ribs is 2.5 m; rigid sheet metal diaphragms with hatch openings are used at 10.0 and 12.5 m spacing. The structure is designed as welded, with five pre-stressed bolt girder mounting contacts made at the construction site and sidewalk supports bolted to the main box girder. High-strength M-20 grade 10.9 bolts with appropriate nuts and washers are provided in the connections. The total weight of the steel bridge structure is approximately 3500 t.

The basic structural material of the bridge is weathering steel grade 12HNNbA, performing as an experimental steel dedicated to bridge applications. It was developed in Poland in the early 1980s by the Institute of Metallurgy of Iron for large dynamically loaded constructions operating at low temperatures. The chemical composition of 12HNNbA was designed as a metallurgical modification of the previously available 10HNAP steel. The chemical composition of 12HNNbA steel is shown in Table 1.

The rust-resistant steel structure of the bridge has been left unpainted, except the first section of the eastern link, which was internally painted in 1998. The steel structure of the bridge was made by the Commune of Paris Shipyard in Gdynia and drifted to the erection site as self-floating items. The eastern section of the bridge (Chyżne-Gdańsk) was opened in June 1990, and the second section was opened two years later, in June 1992. The bridge is located at a sizable distance from major urban centres and industrial facilities. It is not located in the coastal zone. The climatic zone in Poland is defined as moderate (i.e., warm, transitional type). Due to the high frequency of surface moisture on the bridge, structural elements (associated with riverine condensation) and the use of de-icing agents on the road surface, the corrosivity category in which the structure operates can be defined as medium C3, according to ISO 12944-2 [1]. In an environment with corrosivity class C3, the average corrosion rate of weathering steels, according to ISO 9224 [2], is 8.3 < r_av_ < 17 μm/y in the first 10 years of service. The steady-state corrosion rate r_lin_ estimated as the average corrosion rate during the first 30 years for corrosion class C3 is 4.9 < r_lin_ < 10 μm/y.

During the present examination, it was noticed that the bridge had an invalid drainage system for several years, and the zones adjacent to the drainage wells were regularly soaked with the deck water. Moreover, it was observed that the use of calcium chloride (CaCl_2_) as a de-icing agent was a common practice during the wintertime.

The review of the structure for corrosion damage was limited to identifying the locations of such phenomena, measuring the thickness of the box girder plates (bottom flange, webs, upper orthotropic plate, ribs, cross frames, and girder plates), and analysing the condition of the natural patina. The inspection was performed in 2020 by direct observation of the box girder interior and exterior structural components of the bridge. One of the non-destructive methods of assessing the quality of weathering steels and the degree of protection of structures used to assess the state of the patina was the analysis of the phase composition of patina products found on the steel surface with the determination of the proportion of stable and reactive phases, determined by the PAI coefficient [3,4]. By correlating the theoretical knowledge of weathering steel corrosion with corrosion processes occurring on an engineering structure operating in a well-defined corrosive environment, the knowledge of these phenomena can be broadened. An additional benefit of the practical field test presentation is that it provides inspectors with the knowledge of visual assessment of engineering structures and possible corrosion effects of weathering steels.

The patina layer which forms spontaneously on weathering steels has a complex chemical and phase composition [5,6,7]. It is a mixture of various crystalline and amorphous phases, consisting mainly of iron oxides and hydroxides. Among the corrosion products of weathering steel, goethite (α-FeOOH) is the most stable one. Magnetite (spinel, Fe_3_O_4_), hematite (α-Fe_2_O_3_), maghemite (γ-Fe_2_O_3_), ferrihydrite (5Fe_2_O_3_-9H_2_O) and the least stable lepidocrocite (γ-FeOOH) are also components of the oxidised layer. Akaganeite (β-FeOOH) is also observed in the presence of chloride ions [8,9], while the presence of hematite may be related to an SO_2_-rich atmosphere [10,11]. A tight patina layer, which reduces the corrosion rate of steel, forms gradually under the influence of wetting and drying cycles of its surface. Under conditions of prolonged immersion in fresh or sea water, the corrosion rate of weathering steel is the same as that of carbon steel [12]. The absence of a drying cycle inhibits the formation of a protective oxide film, mainly by preventing the transformation of lepidocrocite to goethite [13].

The correlation between the phase composition of the patina on the surface of weathering steels and its stability, which directly affects the corrosion rate, is an important factor in assessing the condition of engineering structures [4]. The corrosion properties of weathering steels are due to the nature of goethite formed in the patina volume, whose densely packed structure of oxygen ions limits the rate of diffusion of other ions [14,15]. The transformation of the most reactive phase in the patina volume (i.e., lepidocrocite into goethite) is included in the Protective Ability Index (PAI) of the oxide layer, calculated from the mass fractions of goethite and lepidocrocite α/γ. The α/γ ratio indicates the reactivity (α/γ < 1) or stability (α/γ > 1) of the protective layer. The transformation of lepidocrocite to stable goethite occurs over time after exposure to weathering, reducing the corrosion rate of the steel. The α/γ ratio is about 1 during the first 5–10 years of exposure and is higher than 2 for patinas of objects exposed to weathering for more than 10 years [16]. A high α/γ ratio provides the desired protective ability of the rust layer to extend the life of weathered steel structures. Hara et al. [3,17] used PAI_α_ and PAI_β_ considering different crystalline phases of patinas to evaluate the protective capacity of the patina layer formed on weathering steel. In these indices, the presence of reactive phases is important because akaganeite (*β*-FeOOH) forms in the presence of chlorides and is easily reduced, which significantly increases the corrosion rate. Magnetite Fe_3_O_4_ as a spinel phase is often formed by the reduction of akaganeite and, through its high conductivity [18], also increases the redox reaction rate, especially when the patina layer remains wet for a long time. PAI_α_ (1) and PAI_β_ (2) are expressed as mass shares of the respective phases included in the patina of weathering steels [3]:

(1) $${\mathrm{PAI}}_{\alpha }=\frac{\alpha }{\beta +\gamma +s}$$

(2) $${\mathrm{PAI}}_{\beta }=\frac{\beta +s}{\beta +\gamma +s}$$

PAI_α_, like PAI, is a criterion for the protective ability of the patina layer, while PAI_β_ determines the corrosion rate of the oxide layer. If PAI_α_ > 1, then the corrosion rate of the steel is less than 0.01 mm/year, while if PAI_α_ < 1, then the corrosion rate can be varied and depends on PAI_β_:

- If PAI_β_ <≈ 0.5, then the corrosion rate is less than 0.01 mm/year,
- If PAI_β_ >≈ 0.5, then the corrosion rate exceeds 0.01 mm/year.

Based on the studies of Hara et al. and Kubzov et al. [3,4], the following patina states can be distinguished:

(1) When most of the components of the oxidised layer on steel are reactive components other than goethite (PAI_α_ < 1) and PAI_β_ >≈ 0.5, the patina does not properly perform its barrier function. The oxidised layer is relatively thick (>400 µm) and may peel or fall off in fragments; with regard to protection type, this type of patina is categorised as non-protective and active.
(2) When most of the components of the oxidised layer on steel are reactive components other than goethite (PAI_α_ < 1) and PAI_β_ <≈ 0.5, it means that there is still a large proportion of γ-FeOOH lepidocrocite in the patina layer, which under suitable atmospheric conditions (dry/wet cycles) will transform into stable goethite, forming a protective layer for steel. This is the case if there is no high chloride concentration in the environment of the rust-resistant steel, and the patina formed in the initial stage of the object’s life is stable for a long time. The patina thickness is usually less than 400 µm. Lepidocrocite is electrochemically active and acts as a cathode [19], so during the initial period of patina formation, when its mass proportion relative to goethite is high, the corrosion rate is above 0.01 mm/year. After the steel is exposed to weathering for a longer period of time, as the proportion of goethite in the patina composition increases, the corrosion rate drops below 0.01 mm/year.
(3) When the patina consists mainly of goethite (PAI_α_ > 1), it is a protective layer and its appearance is uniform and compact.

## 2. Materials and Methods

Seven representative samples were selected for PAI determination based on qualitative and quantitative XRD analysis from each of the interiors of the box girders and the exterior surfaces of the bridge structural components on which the patina appearance differed significantly in colour, texture, and adhesion of corrosion products. X-ray powder diffractometry was used to analyse the phase composition of the natural patina in each section of the structure. For XRD phase analysis, samples were homogenised and ground in an agate mortar and then sieved through a 40 μm mesh sieve. The qualitative analysis of the phase composition of the samples was performed based on the interpretation of diffractograms prepared with an XRD7 X-ray diffractometer from Seifert-FPM. X-ray characteristic radiation Cr K_α_ (λ = 2.291 Å) and V filter were used. The measurements were performed in Bragg Brentano geometry. The analysis was carried out in the 2θ angle range from 15° to 100°, corresponding to the interplanar distance range d_hkl_ from 0.116 to 0.443 nm. Identification of the compounds present in the sample was performed using software from Seifert and Match! software, using the Reference Intensity Ratio (RIR) method and the 2019 ICDD PDF-4+ catalogue data. This method is based on the comparison of intensity scaling factors I/Ic (i.e., ratio of the intensity of the highest peak of a phase to the intensity of the highest peak of corundum standard) for all component phases.

Table 2 shows the main analysed crystalline phases identified in the investigated samples used to calculate the PAI coefficients. The presence of amorphous and non-stoichiometric phases can affect the validity of the quantitative XRD analysis which was performed; however, the determination of PAIs is not based on the assumption that the patina consists entirely of crystalline forms of iron oxides and hydroxides, although the quantitative ratio of selected stable and reactive phases is crucial.

For the analyses performed, the samples were homogenised and ground in an agate mortar and then sieved through a 40 μm mesh sieve. Fourteen samples were analysed and taken from the facility at locations with varying degrees of steel surface corrosion.

Based on the content of the various crystalline phases in the volume of patinas found inside and outside the box girders, PAI_α_ and PAI_β_ were determined, and the character of the protective layer (active/passive) and thus the degree of protection of the structure was evaluated.

Besides the qualitative and quantitative phase analysis, the characterisation of the corrosion products of the weathering steel was limited to a visual assessment of the colour and the adhesion of the rust layer (compact and strongly bonded layer/layer that peels off spontaneously or with little force and a sharp tool). Thickness measurements of the patina layer on ferromagnetic substrates using a thickness gauge based on magnetic induction are subject to high uncertainty because some of the patina components show strong or weak ferromagnetic properties (magnetite/goethite). Therefore, the thickness of the patina layer was not determined. No direct measurements were made of the thickness of the peel-off layer because it was not possible to assess whether the detachment occurred at the substrate or in the volume of the rust layer.

## 3. Results and Discussion

**Figure 1:**
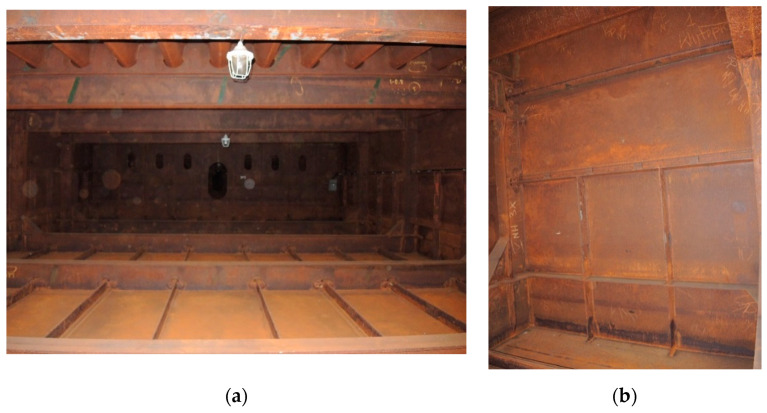
Typical appearance of weathering steel plates in the interior of a box girder (**a**) and on the side wall of a box girder (**b**).

Figure 1a,b shows the typical appearance of weathering steel sections in the interior of the box girder and on the web. Inside the box girder, the colour of the patina on individual parts of the structure is varied. On the side wall of the girder, the patina ranges from light brown (rusty) to dark brown. Darker colours occur especially in the zone of the bottom of the structure and the lower parts of the webs, probably due to the zone of immersion of the section (up to approximately 90 cm) during floating of the elements by the river. Besides these places, patina discolouration in the form of smudges occurs mainly in the zone of the orthotropic section plate. Table 3 shows the appearance of patinas observed on various structural members of the bridge inside the box girders, along with characteristics regarding the colour and adhesion of the protective layer. According to the literature [3,4,20,21], at the initial stage of corrosion of weathering steel, the patina formed is light brown in colour and, as the phase transformations continue, the patina layer darkens to a dark brown colour. The thickness of the patina increases gradually to hundreds of micrometres. If the patina layer is too thick, it tends to peel off and the steel surface is not protected.

**Figure 2:**
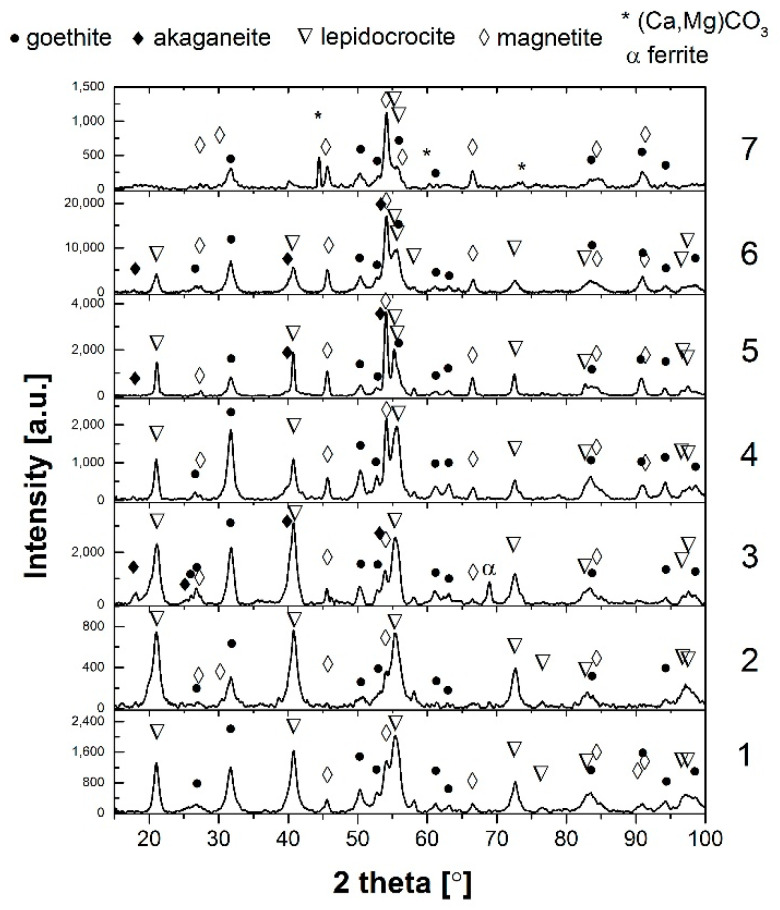
X-ray diffractograms of patinas from the analysed areas shown in Table 4, with qualitative analysis results.

Figure 2 shows X-ray diffractograms of patinas formed on the analysed areas of the weathering steel with the results of qualitative analysis. Table 4 shows the mass fraction of individual crystalline phases in the patina volumes and the calculated PAIs. The rust layer formed on the weathering steel inside the boxes is mainly composed of α-FeOOH (goethite), γ-FeOOH (lepidocrocite) and spinel Fe_3_O_4_ (magnetite). Additionally, β-FeOOH (kageneite) was also observed in samples 5 and 3, (Ca, Mg)CO_3_ was present in sample 7 and some ferrite traces were observed in sample 3. Hara et al. [17,22] found that β-FeOOH and Fe_3_O_4_ can be formed in the presence of chlorides and magnetite Fe_3_O_4_, as a spinel phase is often formed by the reduction of kageneite, especially when the patina layer remains wet for a long time.

Based on the calculation of the PAI_α_ coefficient, it can be concluded that only the reddish-brown patina from sample 4 (taken from the middle zone of the wall of the web), according to [3,4], meets the PAI_α_ > 1 criterion and is an active protection for the weathering steel. The patina consists mainly of goethite (59 wt.%) and the passive layer is stable.

**Figure 3:**
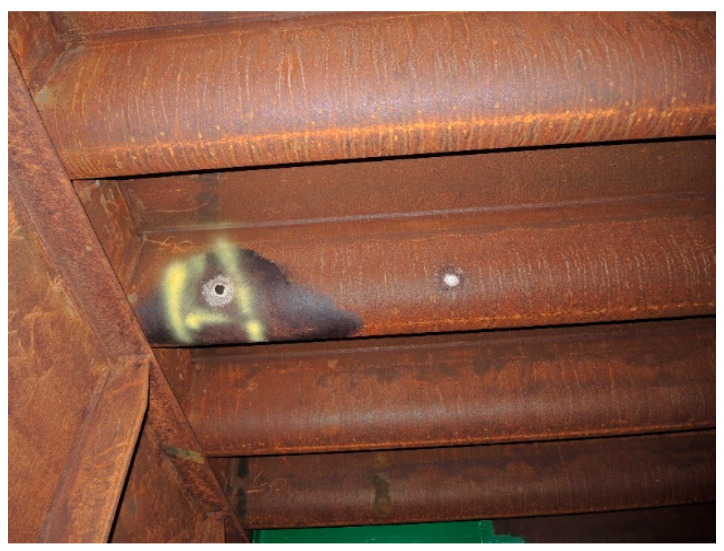
Perforation of an orthotropic plate rib at a patina area containing a high mass fraction of kageneite, as in sample 5.

PAI_α_ < 1 and PAI_β_ >≈ 0.5 indicates that the majority of the patina components is composed of reactive phases other than goethite, which corresponds to samples 5, 6 and 7. In this condition, the patina layer typically reaches a thickness of > 400µm [3,4] and may peel or fall off in fragments, as observed in samples 6 and 7. Furthermore, the predominant components of the patina in sample 7 are phases other than goethite, such as magnetite and calcium magnesium carbonate (Ca, Mg) CO_3_, hence the unusual red/orange colour of the patina. When most of the components of the kagenei layer on the steel are reactive components other than goethite (PAI_α_ < 1) and PAI_β_ >≈ 0.5, the patina does not properly perform its barrier function. Sample 5 was taken from the bottom of the ribs of an orthotropic plate and exhibits a very high content of kageneite at about 40% by weight, which forms in the presence of chloride ions [8,9]. This type of patina was formed due to leakage in the bridge insulation and water pooling inside the ribs. Under long-term immersion in fresh or sea water, the corrosion rate of weathering steel is the same as for carbon steel [12]. Upon further inspection of the bridge, it was observed that in areas of black patinas at the bottom of the ribs, corrosion of the steel locally occurs to the point of perforation with distinct salt crystals (mainly hydrated sodium carbonate Na_3_H (CO_3_)_2_—2H_2_O) around the perforation, as shown in Figure 3.

PAI_α_ < 1 and PAI_β_ <≈ 0.5 indicates that there is still a large proportion of γ-FeOOH lepidocrocite in the patina layer, as in the case of samples 1, 2 and 3. Lepidocrocite under suitable atmospheric conditions (dry/wet cycles) will transform into a stable goethite if there is no high chloride concentration in the environment of the weathering steels. Corrosion products with PAI_α_ < 1 and PAI_β_ <≈ 0.5 were observed on the inner girders of bridges located in rural mountainous areas (with short wet periods) after 7 years of service [17]. This type of corrosion product layer, due to the slowed redox reaction, is referred to as inactive, in which the corrosion rate depends on the mass ratio of goethite and lepidocrocite phases (α/γ) and the γ → α transformation rate, which is high during the initial patina formation period and decreases with time.

**Figure 4:**
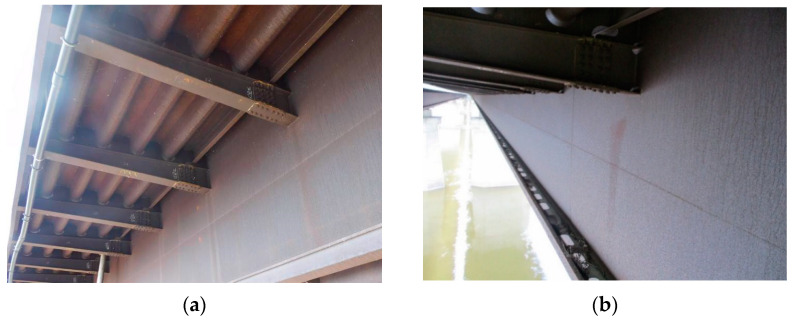
The bridge structure in the support part (**a**) and the typical appearance of plates made of weathering steel on the external side of a box girder (**b**).

Figure 4a,b shows the deck structure in the support section and the typical appearance of the weathering steel plates on the outside of the box girder. In contrast to the patina colours found inside the box girder, the patina formed under external conditions is dark brown and brown. A layer of patina of varying thickness and smudges was observed on the girder webs. Table 5 shows the appearance of patinas observed on various structural members of the bridge on the outside of the box girders, with characteristics regarding the colour and adhesion of the protective layer.

**Figure 5:**
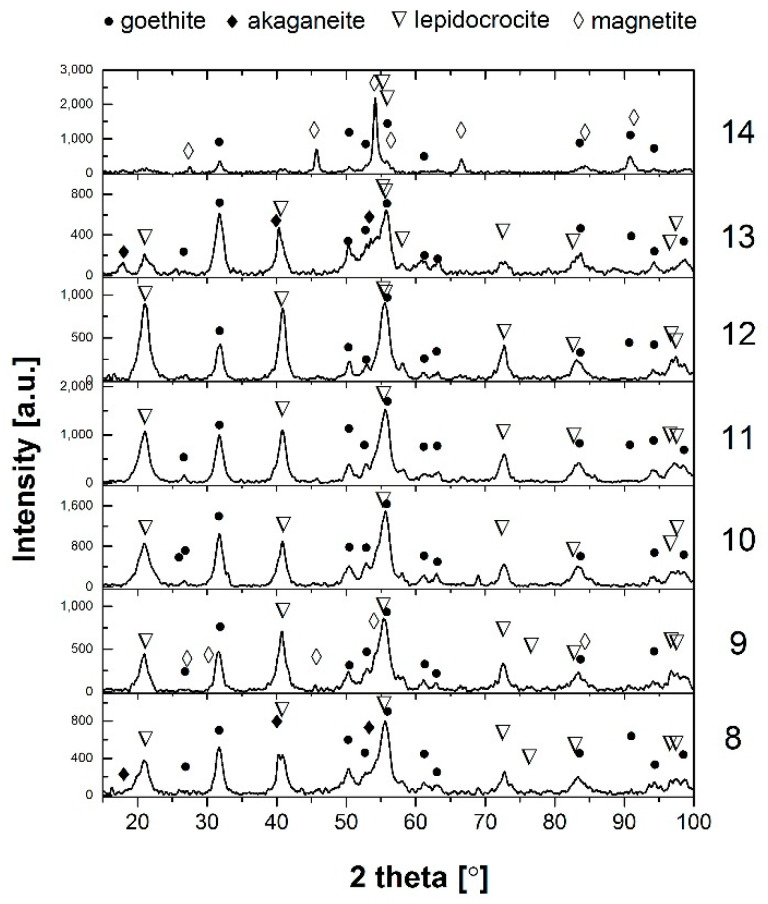
X-ray diffractograms of patinas from the analysed areas shown in Table 6, with qualitative analysis results.

Figure 5 shows X-ray diffractograms of patinas from the analysed areas with the results of qualitative analysis. Table 6 shows the weight fraction of each crystalline phase in the patina volumes and calculated PAIs. The rust layer formed on the weathering steel on the outside of the boxes is mainly composed of α-FeOOH (goethite) and γ-FeOOH (lepidocrocite). Additionally, β-FeOOH (akaganeite) was observed in samples 8 and 13, and spinel Fe_3_O_4_ (magnetite) was present in samples 9 and 14. According to the literature [23,24], the development of corrosion products on weathering steels strongly depends on the local microclimate nearby the bridge structure, as some surfaces can be rained down.

Based on the calculation of the PAI_α_ coefficient, patina samples for which PAI_α_ > 1 can be distinguished. These are samples 8, 10, 11 and 13. According to the literature [3,4], this type of patina actively protects weathering steel and the passive layer is stable. All these samples of corrosion products come from the outer surfaces of the dark brown web, with visible smudges forming on the surface due to water condensation. On sample 13, these smudges are highly visible in the form of brighter and shinier corrosion products, and the phase composition analysis indicates an increased amount of akaganeite with respect to the other samples. The high content of akaganeite in sample 13 is caused by a design mistake in the drainage of the bridge deck slab, which results in the flooding of the web and the bottom flange of the main girder from water from the bridge deck.

Sample 8, despite the predominance of goethite in its phase composition, also shows a high content of akaganeite and is additionally characterised by a greater thickness and weaker adhesion to steel than the other patina types. Samples 10 and 11 do not have any reactive crystalline phases other than lepidocrocite, hence PAI_β_ cannot be determined for these samples.

PAI_α_ < 1 and PAI_β_ >≈ 0.5, typical for a patina composed predominantly of reactive components other than goethite, corresponds to sample 14. The patina layer is very thick (>400 µm), peeling or falling off spontaneously in some areas. The predominant patina component in sample 14 is magnetite, and the patina does not function properly as a protective barrier.

PAI_α_ < 1 and PAI_β_ <≈ 0.5 was found for sample 9, which has comparable goethite and lepidocrocite contents. Lepidocrocite under suitable atmospheric conditions (dry/wet cycles) can transform into stable goethite if there is no high chloride concentration in the environment [4,23]. The patina from sample 9 does not visually differ from the other samples taken from the outer surfaces of the web (samples 10 and 11), but in contrast to them, it contains an increased content of magnetite in its phase composition—11 wt.%.

In contrast to the patina samples taken from the interior of the box girders, the samples taken from the exterior webs have patinas that do not have any reactive crystalline phases other than lepidocrocite in their composition, and the PAI_β_ coefficient cannot be determined for them. These are samples 10 and 11, mentioned before, on which the patinas have a protective function, and sample 12, taken from the cross member. Taking into account the very good adhesion of the patina to steel, its uniform colour and fine crystalline nature, as well as the predominance of lepidocrocite over goethite, it can be concluded that the rate of patina formation on these elements is the lowest in the entire structure. This type of patina crystalline state is typical of weathering steel operating under mainly dry conditions with short wetting periods [3,4,25,26]. Lepidocrocite is electrochemically active and acts as a cathode [19], so in the initial period of patina formation, its mass fraction relative to goethite is high; the γ→α transformation rate is also high, and it decreases with time.

## 4. Conclusions

As a result of the observations and analyses performed, it was found that the steel structure did not undergo passivation in a typical manner, as described in the literature [3,4,19]. The colouring of the natural patinas on the girder surfaces is clearly related to the specific environmental conditions; the external surfaces are dark brown, whereas the inside of the girders is characterised by much lighter shades of brown. Inside the boxes, a greater differentiation of patina colours on individual fragments of the structure is also visible. The darker colours are particularly found in the underside areas of the structure and the lower parts of the webs, which is probably related to the immersion zone of the section (up to about 90 cm) when the components were floated down the river. Outside these areas, patina staining and discolouration occurs mainly in the orthotropic plate zone. Variations in the colour of the natural patina on the exterior surfaces occur mainly in areas where staining occurs.

Based on the calculated PAI coefficient values, it can be concluded that a patina composed of active and inactive crystalline phases occurs on structures that are next to each other. The corrosion products formed on parts of the steel surface reduce the corrosion rate in a barrier function (PAI_α_ > 1) and in an electrochemically active function (PAI_α_ < 1 and PAI_β_ <≈ 0.5). Alternatively, most of the components of the oxidised layer on steel are reactive components other than goethite (PAI_α_ < 1 and PAI_β_ >≈ 0.5), and the patina does not properly perform its protective function.

First of all, during their formation period, patinas must be subjected to cyclic periods of wetting and drying in order for electrochemical processes to initiate their development. Prior observations and research carried out by the Road and Bridge Research Institute (Warsaw, Poland) showed that box-type structures do not provide the desired conditions during the patina formation period, which is also the case in the analysed example. In the closed spaces of the orthotropic deck plate ribs, where damage has occurred, the corrosion products of the steel are active in nature, but the high proportion of reactive phases, including akaganeite associated with the presence of chlorides in the surrounding atmosphere, is significant, and the degree of protection of the steel is insufficient. The aggressive nature of chlorides and moisture causes accelerated corrosion of the weathering steel through the transformation of stable goethite to akageneite [8,9,27]. The content of akaganeite was significantly higher (relative to the other samples) in the samples taken at the dark grey spots on the ribs and near the white spots at the steel perforation site. This is consistent with the observations of Nishimura et al. [28], who showed that Cl-rich atmospheres very rapidly accelerate the corrosion processes of weathering steels because the high concentration of Cl- ions leads to the formation of akaganeite, which can be reduced electrochemically in the corrosion process. Morcillo et al. [29] showed that the atmospheric environmental conditions necessary for the formation of akaganeite are an annual average RH of approximately 80% or higher, and simultaneously, an average annual chloride ion deposition rate of approximately 60 mg/m^2^/day or higher. Chloride ions, whose high concentrations and lowered pH (4–6) give rise to akaganeite formation, also accumulate in areas of the structure heavily exposed to debris accumulation.

Based on the PAIs, it was also noted that despite many years of age, in several areas of the girder interior, the patinas have not yet reached a stable state, and a phase transformation of lepidocrocite to goethite (γ → α transformation) is still occurring. This may be related to the specific conditions inside the boxes, without wet/dry cycles, affecting the rate of γ → α phase transformation in the patina volumes. Despite the thirty years of service, in many places inside the structure, the patina formation process has not finished, and the full effect of structural steel protection has not been achieved (samples 1, 2 and 3).

Based on the PAIs, a similar patina condition was also observed on the outer surfaces of the web (samples 9 and 12), despite the visual similarity to the sample taken from the outer wall of the web (sample 10), which according to the determined PAI, protects the structure in a barrier-like manner. The only difference between samples 9 and 10 concerns the magnetite content, which was significantly higher in sample 9. In the remaining cases, the external conditions of the weathering steel (mainly dry/wet cycles) has allowed for the formation of a continuous patina layer with the appearance and character of a passive layer adequate to the service life.

## Figures and Tables

**Table 1:** Content of alloying elements in weathering steel 12HNNbA (balance: Fe).

Element	Percentage by Weight
C	0.09–0.14
Cr	0.70–1.10
Mn	0.60–1.00
Ni	0.30–0.60
Si	0.30–0.60
Cu	0.30–0.55
Al	0.06–0.15
Nb	0.015 –0.035
P	≤0.035
S	≤0.035

**Table 2:** Major crystalline phases identified in the patina samples analysed.

Crystalline Phase	JCPDS Card Number	Major Peak Positions d_hkl_/2θ-λ_Cr_ [°]		
Goethite: α-FeOOH	04-015-8332	4.188/31.73	2.452/55.66	2.695/50.28
Akageneite: β-FeOOH	04-018-6388	7.375/17.86	3.309/40.48	2.538/53.63
Lepidocrocite: γ-FeOOH	04-014-3986	6.255/21.09	3.291/40.71	2.467/55.30
Magnetite: Fe^2+^Fe^3+^_2_O_4_	04-007-2718	2.525/53.92	1.481/101.3	2.961/45.49

**Table 3:** Appearance of the patina layer observed on different structural elements of the bridge inside the box, together with characteristics regarding the colour and adhesion of the passive layer.

Sample No.	Appearance of the Patina Layer	Characteristics of the Passive Layer
1	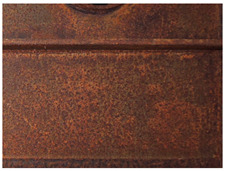	homogeneous, rust-brown patina on the cross beam with good adhesion, locally with darker fragments of lower adhesion
2	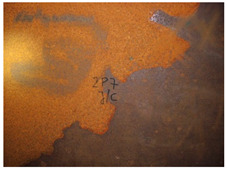	homogeneous orange patina on the girder web, with fine crystalline structure and good adhesion
3		homogeneous brown patina layer (underneath the orange-yellow layer) on the girder web, with fine crystalline structure and good adhesion
4	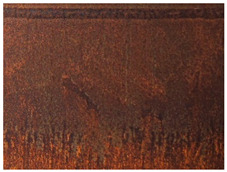	patina with good adhesion on the girder web, reddish-brown in colour, fading into dark brown areas with reduced adhesion (spontaneous flaking in some areas)
5	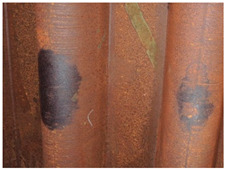	dark brown/black corrosion products on the ribs of the orthotropic plate, compact layer, with glossy appearance and good adhesion
6	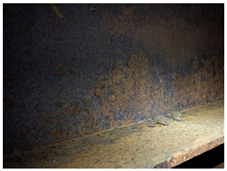	dark-brown flaking/spontaneously detaching layer of patina on girder webs
7	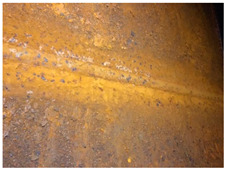	yellow-brown peeling and corrosion products falling off on the girder web

**Table 4:** Mass fractions of specific crystalline phases in the patina volumes and calculated PAIs.

Sample No.	Content of Crystalline Phases wt.%				PAI_α_	PAI_β_
	α-FeOOH	β-FeOOH	γ-FeOOH	Spinel Phase		
				s-Fe ^2+^Fe ^3+^_2_O_4_	α/	(β +s)/
	Goethite	Akaganeite	Lepidocrocite	Magnetite	(β + γ + s)	(β + γ + s)
1	45	0	38	17	0.8	0.3
2	32	0	61	7	0.5	0.1
3 *	34	7	50	5	0.5	0.2
4	59	0	23	18	1.4	0.4
5	32	40	24	4	0.5	0.6
6	41	9	18	32	0.7	0.7
7 *	25	0	0	50	0.5	1.0

* samples containing crystalline phases other than specified in the table.

**Table 5:** Appearance of patinas observed on various bridge structural components on the outside of the boxes and characteristics regarding colour and adhesion of the passive layer.

Sample No.	Appearance of the Patina Layer	Characteristics of the Passive Layer
8	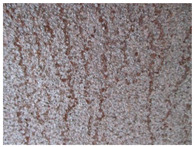	dark brown thick layer of patina with visible smudges on the outer surfaces of the web, locally coarse crystalline, with good adhesion
9	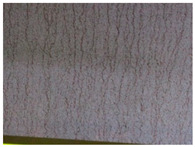	dark brown patina with visible smudges on the outer surfaces of the web, with good adhesion
10	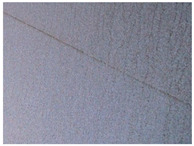	dark brown patina with visible smudges on the outer surfaces of the web, with good adhesion
11	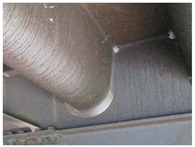	dark brown patina with visible smudges on the ribs of the deck plate, with good adhesion
12	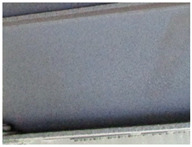	dark-brown homogeneous patina on the cross beam, fine crystalline, good adhesion
13	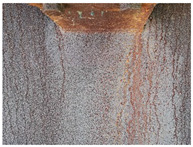	light brown shiny layer of patina with visible smudges on the web of the girder, locally coarse crystalline, with varying adhesion
14	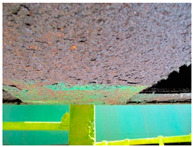	very thick layer of corrosion products, falling off in fragments, incoherent, on the cross beam of the girder

**Table 6:** Mass fractions of specific crystalline phases in the patina volumes and calculated PAIs.

Sample No.	Content of Crystalline Phases wt.%				PAI_α_	PAI_β_
	α-FeOOH	β-FeOOH	γ-FeOOH	Spinel Phase		
				s-Fe^2+^Fe^3+^_2_O_4_	α/	(β + s)/
	Goethite	Akaganeite	Lepidocrocite	Magnetite	(β + γ + s)	(β + γ + s)
8	56	30	14	0	1.3	0.7
9	44	0	45	11	0.8	0.2
10	54	0	46	0	1.2	-
11	49	0	51	0	1.0	-
12	35	0	65	0	0.5	-
13	64	15	21	0	1.8	0.4
14	24	0	12	64	0.3	0.8

## Data Availability

The results of the study are not placed in any publicly archived datasets.
